# Supplementary material for: Neither alpha-synuclein fibril strain nor host murine genotype influences seeding efficacy
Source: NPJ Parkinsons Dis. 2024 May 21;10:105. doi: 10.1038/s41531-024-00679-1 (PMC11109094; doi:10.1038/s41531-024-00679-1)
Supplement: Supplementary file 1 — Supplemental Material [file 41531_2024_679_MOESM1_ESM.pdf]

Supplementary Information for: Neither alpha-synuclein fibril strain nor host murine genotype influences seeding efficacy.

Sara Walton[1], Alexis Fenyi[2], Tyler Tittle[3], Ellen Sidransky[4], Gian Pal[5], Solji Choi[3], Ronald Melki[2], Bryan A. Killinger[3], and Jeffrey H. Kordower[1]

[1]ASU-Banner Neurodegenerative Disease Research Center and School of Life Sciences, Arizona State University, Tempe, AZ, USA.

[2]Institut Francois Jacob (MIRCen), CEA and Laboratory of Neurodegenerative Diseases, CNRS, Fontenay-Aux-Roses Cedex, France.

[3]Graduate College, Rush University Medical Center, Chicago, Illinois 60612

[4]Medical Genetics Branch, National Human Genome Research Institute, National Institutes of Health, Bethesda, Maryland, USA.

[5]Department of Neurology, Division of Movement Disorders, Rutgers - Robert Wood Johnson Medical School, New Brunswick, NJ, USA.

Corresponding author: Bryan Killinger

Table of Contents

Supplementary Methods
Supplementary figure 1. Characterization of patient brains homogenates.
Supplementary figure 2. Amplification of pathogenic αSYN from patient's brain homogenates by PMCA.
Supplementary figure 3. In vitro characterization of patient-derived αsyn PFFs.
Supplementary figure 4. PSER129 abundance in mice seeded with de novo PFFs or PFF
strains.
Supplementary figure 5. BAR target enrichment.
Supplementary figure 6. Summary of BAR-PSER129 Enriched Proteins.
Supplementary figure 7: Odor retention test in GBA1D409V/D409V mice treated with different alpha-synuclein human pre-formed fibrils (HuPFFs).
Supplementary figure 8: Odor retention test in wild type mice treated with different alpha-synuclein polymorphs (strains).
Supplementary figure 9: Digging odor test in GBA1D409V/D409V mice treated with different alpha-synuclein human pre-formed fibrils (hPFFs),
Supplementary figure 10: Rotarod test in GBA1D409V/D409V mice treated with different alpha-synuclein human preformed fibrils (hPFFs).
Supplementary figure 11: Odor threshold test in GBA1D409V/D409V mice treated with different alpha-synuclein human pre-formed fibrils (hPFFs).
Supplementary figure 12: Odor threshold test in wild type mice treated with different alpha-synuclein polymorphs (strains) extracted from the frontal cortex. HOM1 and HOM2 are GBA-hPFFs
Supplementary figure 13: Odor threshold test in wild type mice treated with different alpha-synuclein polymorphs (strains) extracted from the cingulate cortex.
Supplementary figure 14. Comparison of PFF spread in WT and GBA1D409V KI mice.
Supplementary figure Table 1. Case information.
Supplemental References

Supplemental Methods

Odor retention test

The odor retention test assesses short-term olfactory memory using pairs of odorants and is based on methods adapted from previous work [1, 2]. Before the first test, mice were pre-habituated to the setup for 5 min with empty cartridges in a clean mouse cage without bedding. During the test (performed in a blinded manner), the mice were exposed to two separate cartridges at a time that each contained a paper swab with an odorant infused. During the first trial (Acquisition), mice were exposed to the same unfamiliar odorant in the two cartridges. After 6, 16, or 30 min, mice were put through a second trial (Recall) that contained a cartridge containing the same odorant as the first trial, and a second cartridge with a novel odorant. The preferred odor was recorded and the preference was calculated for the recall trial. Three pairs of odorants were used (familiar and novel odor) at supraliminal concentrations. The chosen odorants were known for having equal hedonicity among each pair [1,3] (-) limonene (diluted 1:10) and (+) carvone (diluted 1:10); Amylacetate (1:100) and Anisol (1:100); and propyl acetate (1:10) and Pentanal (1:10). These odorants were diluted in mineral oil. The use of each odorant from the pair as familiar or novel odor was randomized. Data was expressed as a preference for the novel odor. The mean preference for novel odor for each group during the recall trial was analyzed by one-sample Student’s t test

compared with the chance level of 50% (Prism 6.0; GraphPad). The odorants used were purchased from Sigma-Aldrich, TCI America, or provided by A. Didier (Lyon Neuroscience Research Center, Lyon, France).

Odor Threshold

The animals were placed a clean mouse cage without bedding. The test was done individually and each mouse was habituated to the setting during a pre-habituation trial with a cartridge that contained a paper swab soaked in mineral oil (MO). Each mouse was then exposed to a paper swap soaked in mineral oil for three 50s trials with a 5 min inter-trial interval (habituation phase, MO). The animals were then presented with a paper swab soaked in an odorant (propionic acid) at increasing concentrations ($1{:}10^6$, $1{:}10^4$, and $1{:}10^3$, detection phase). in mineral oil. During each trial, the investigation time, defined as the duration of active sniffing with the nose placed less than 1 cm away from the cartridge. Mice that did not investigate the mineral oil during the first habituation trail were excluded. The mean investigation time per trial was calculated and anylyzed by one-way ANOVA with repeated measures across trials for each group and time point. This was done for the habituation phase first which is critical for being able to interpret the results from the following detection phase, and then for the detection phase. This was followed by a Sidak post-hoc test (more conservative than Fisher LSD post-hoc test) to compare MO1 to MO2, MO3, and MO3 to odorant concentrations $1{:}10^6$, $1{:}10^4$, and $1{:}10^3$ using Prism 6.0 (GraphPad Software).

Rotarod

The rotarod test was used to assess motor learning, coordination, and balance in the mice (MED-Associates). Each mouse was given a training session (four 5- min trials, 5 min apart) to acclimate them to the rotarod apparatus. During the test period (1 hr later), each mouse was placed on the rotarod with increasing speed, from 4 rpm to 40 rpm in 300 sec. The latency to fall off the rotarod with in this time period was recorded. Each mouse received two consecutive trials and the mean latency to fall was used in the analysis.

Digging Test

Prior to testing animals were fasted overnight. Fasting did not to exceed 24h. A clean mouse cage (15 cm x 3.25 cm x 3.13 cm) was filled with 3 cm of bedding and an appetitive stimulus of sweetened cereal (Cinnamon Toast Crunch) buried 0.5 cm below the bedding and along the perimeter of the cage. The animal were monitored for 5 mins or until the animal found the food treat (latency to find treat), at which time the session was completed. Once the session was completed, the animals were returned to their home cage and food returned. If the mouse found the treat, it was allowed to eat it. If the treat was not found within 5 mins the mouse placed back in its home cage and the treat was removed. The bedding was changed between mice.

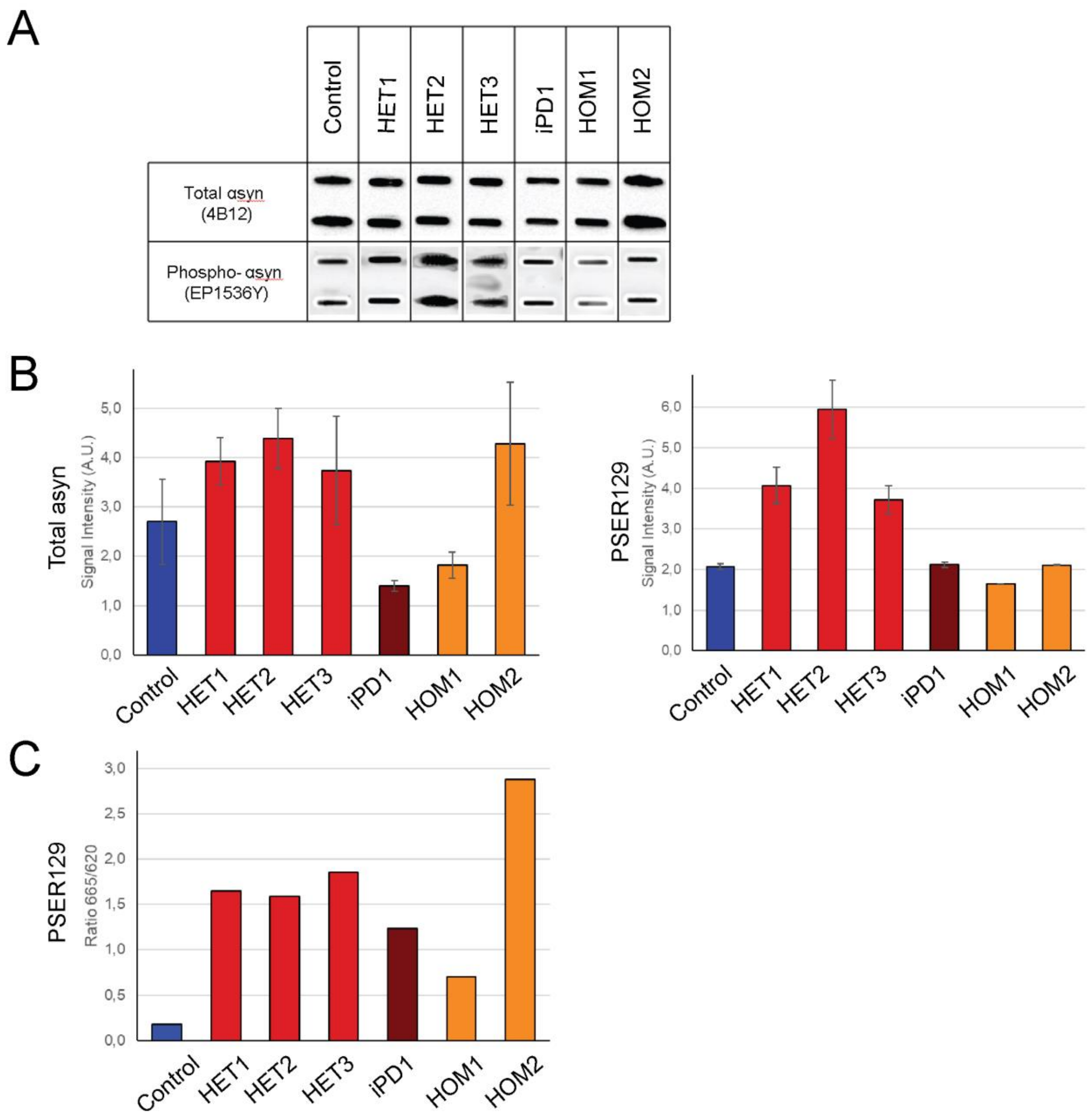

Figure S1. Characterization of patient brains homogenates.

(A) The amount of total and phosphorylated αsyn in the different brain homogenate was determined using a filter retardation assay. 50µL of brain homogenates (1% W:V) were filtered in duplicate on nitrocellulose membrane and probed with 4B12 (total αsyn) or EP1536Y (phosphorylated αsyn). (B) Quantification of total and phosphorylated αsyn in the different brain homogenate presented in panel A, bars represent ±SD. (C) The amount of pathogenic phosphorylated αsyn in the different brain homogenates (2,5% W:V) was quantified using the cisbio FRET assay following the manufacturer's recommendations.

Supplementary Figure 1

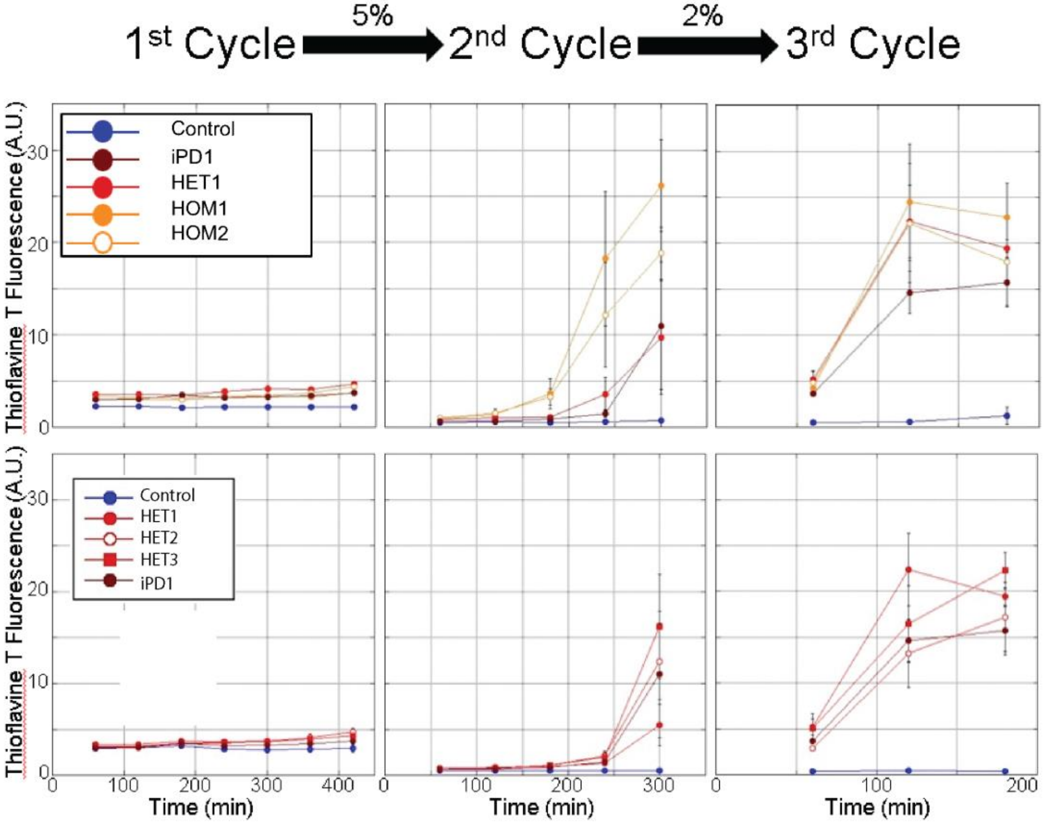

Figure S2. Amplification of pathogenic αSYN from patients brain homogenates by PMCA. PMCA was performed on human brain homogenates (2% (W:V) for the 1st cycle, the indicated amounts (V:V) for the next cycles, in PMCA buffer containing monomeric αsyn (100 µM). The amounts of brain homogenates and PMCA-amplified assemblies used in each amplification reaction were optimized through several trials to minimize the de novo aggregation of αsyn under our experimental conditions. The time at which an aliquot from one amplification reaction was withdrawn for a subsequent amplification reaction was also optimized to avoid the formation of de novo of αsyn fibrillar assemblies. The curves represent an average of four replicates ± SD

Supplementary Figure 2

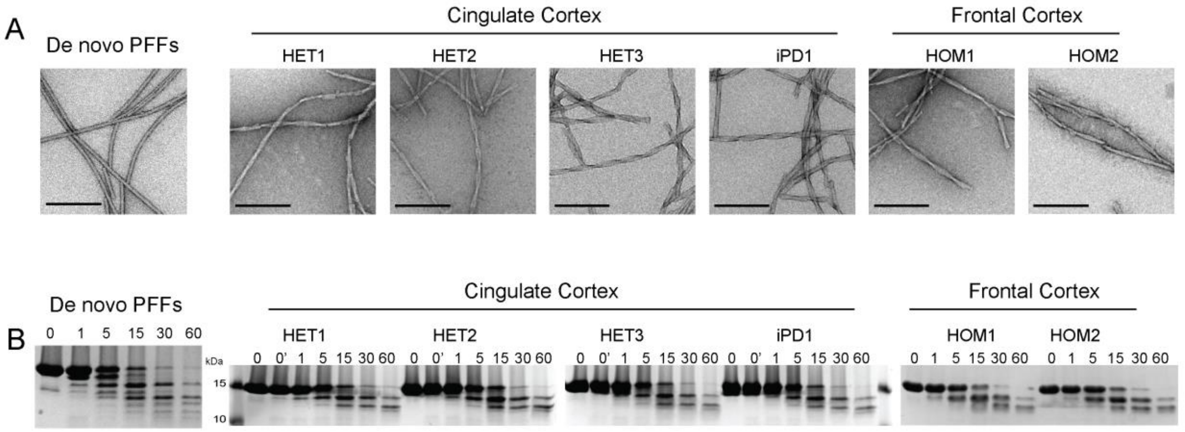

Figure S3. In vitro characterization of patient-derived αsyn PFFs.
(A) Electron micrographs of of patient-derived αsyn PFFs obtained after the 4th cycle of amplification by PMCA and de novo generated PFFs. Scale bar = 200 nm. (B) Limited proteolytic patterns of the different strains. Monomeric αsyn concentration is 100 µM. Proteinase K concentration is 3.8 µg/ml. Samples were withdrawn from the reaction before PK addition (lane most to the left), immediately after PK addition (second lane from left) and at time 1, 5, 15, 30 and 60 min from left to right in all panels. PAGE analysis was performed and the gels were stained with Coomassie blue. The position of the molecular weight markers 15 and 10 kDa is indicated on the left.

Supplementary Figure 3

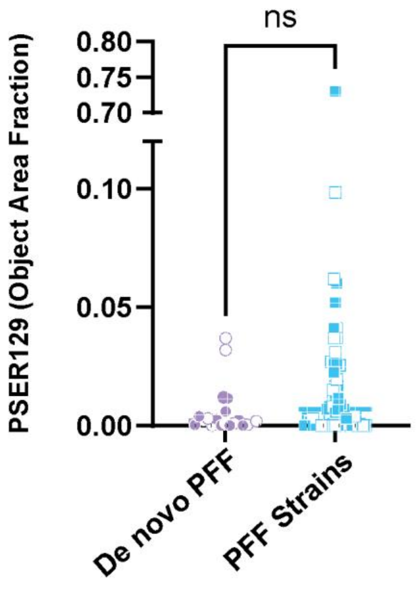

Figure S4. PSER129 abundance in mice seeded with de novo PFFs or PFF strains. ns= non-significant using Unpaired Two-tailed t test.

Supplementary Figure 4

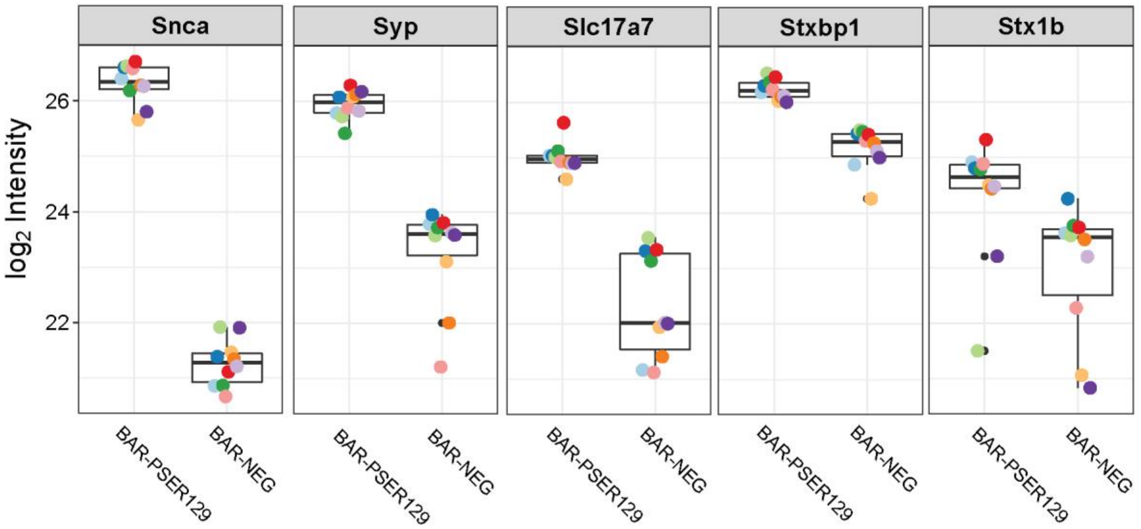

Figure S5. BAR target enrichement. .Raw LC-MS/MS data files were analyzed by Maxquant and LFQ-Analyst (Shah AD, Goode RJA, Huang C, Powell DR, Schittenhelm RB. LFQ-Analyst: An easy-to-use interactive web-platform to analyze and visualize proteomics data preprocessed with MaxQuant. DOI: 10.1021/acs.jproteome.9b00496). Two experimental groups were compared, BAR-Neg and BAR-PSER129. Boxplots of abundance show selected significant proteins including BAR target asyn (SNCA), and several known presynaptic vesicle/SNARE proteins thought to be closely associated with asyn. Results demonstrate high enrichment of asyn and the presynaptic compartment using BAR-PSER129. SYP = Synaptophysin, Slc17a7 = Vesicular glutamate transporter 1, Stxbp1 = Syntaxin binding protein 1, Stx1b = Syntaxin-1B1.

Supplementary Figure 5

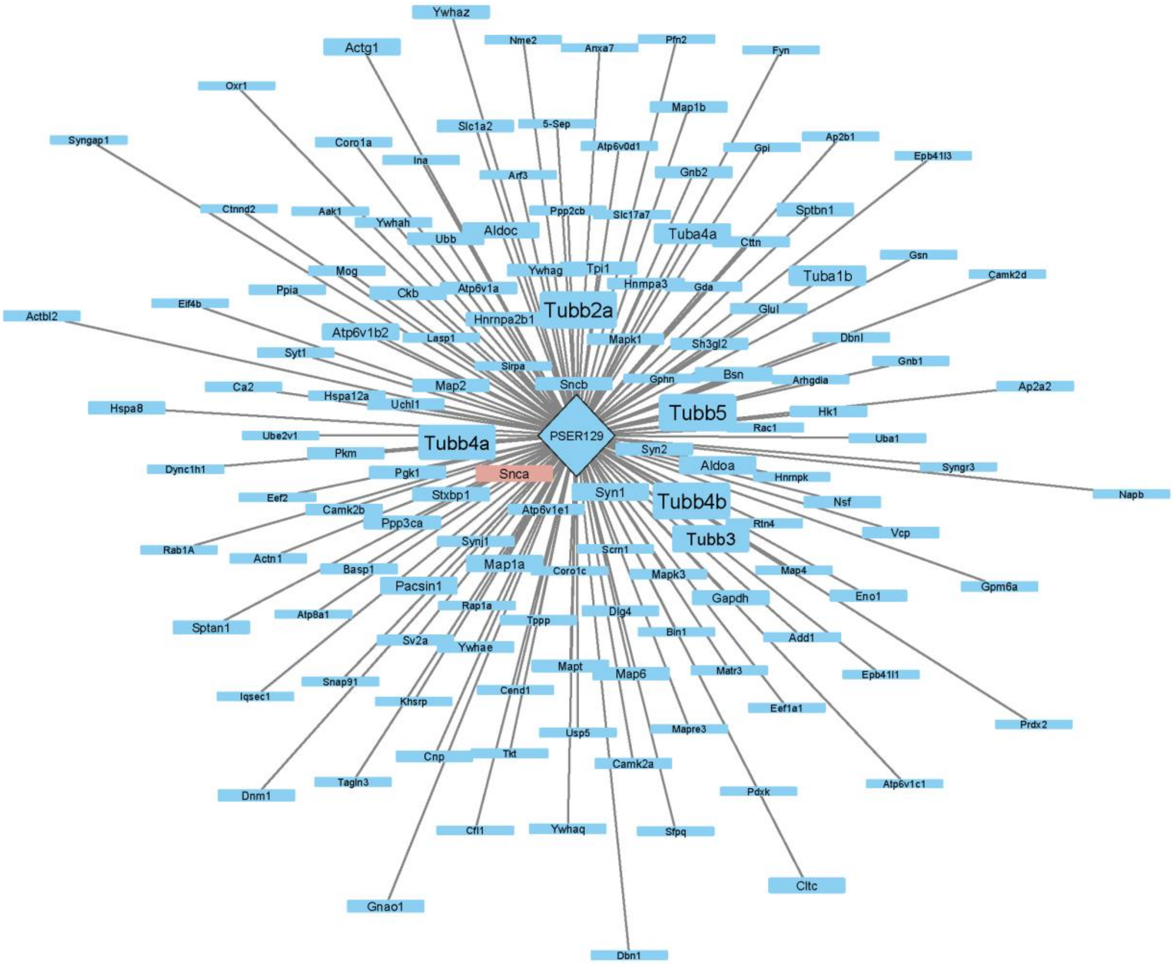

Figure S6. Summary of BAR-PSER129 Enriched Proteins. Enriched proteins included in Fig. 5 were plotted using cytoscape and perfusion force directed layout. Node and text size is directly proportional to difference for total spectra between BAR-PSER129 and BAR-NEG for each protein. The edge length, thus distance from center node "PSER129" is directly proportional to the corresponding q-value for each enriched protein. Alpha-synuclein's node position is highlighted red.

Supplementary Figure 6

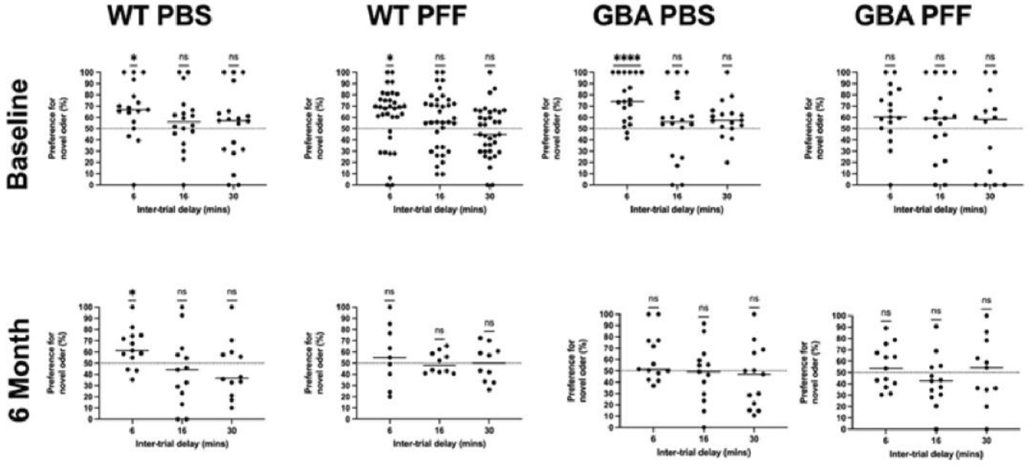

Figure S7: Odor retention test in GBA1$^{D409V/D409V}$ mice treated with different alpha-synuclein human pre-formed fibrils (HuPFFs). At baseline odor retention, the WT mice injected with PBS and HuPFFs and the GBA1$^{D409V/D409V}$ mice injected with PBS showed preference for the novel odor up until 6 mins. This suggest that they remembered the familiar odor for 6 mins, but did not remember it at 16 or 30 mins after initial exposure. The GBA1$^{D409V/D409V}$ mice injected with HuPFFs did not show preference for the novel odor at any time point, suggesting that they did not remember the familiar odor from the start.

Supplementary Figure 7

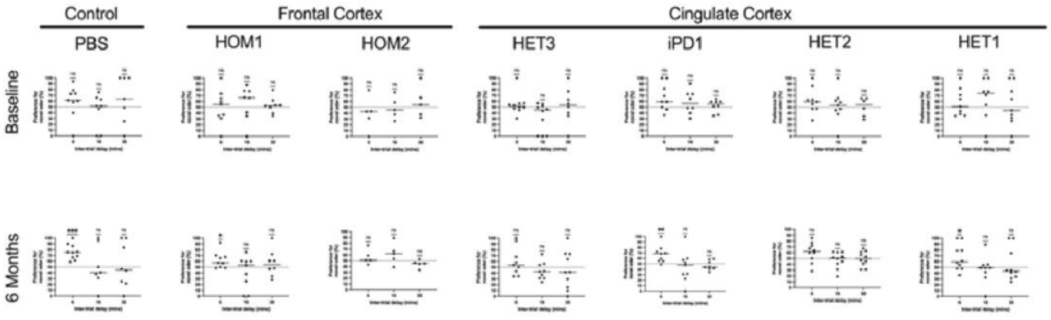

Figure S8: Odor retention test in wild type mice treated with different alpha-synuclein polymorphs (strains). The WT mice injected with PBS, GBA-HuPPFs (HOM1, HOM2, HET1, HET2, HET3), or iPD-HuPFFs (iPD1) did not show a preference for the novel odor during the baseline test. At 6 months, mice treated with B19 and GBA1 variant fibrils had a preference for the novel odor up until 6 mins after the initial exposure. This data suggests that, although there was some recognition at 6-month time points, short-term olfactory memory was impaired in the GBA-HuPPF and iPD-HuPFF injected mice.

Supplementary Figure 8

A

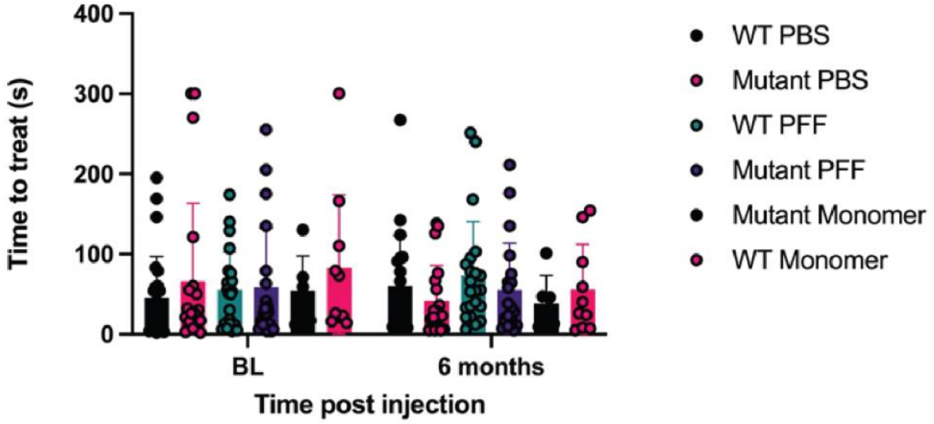

B

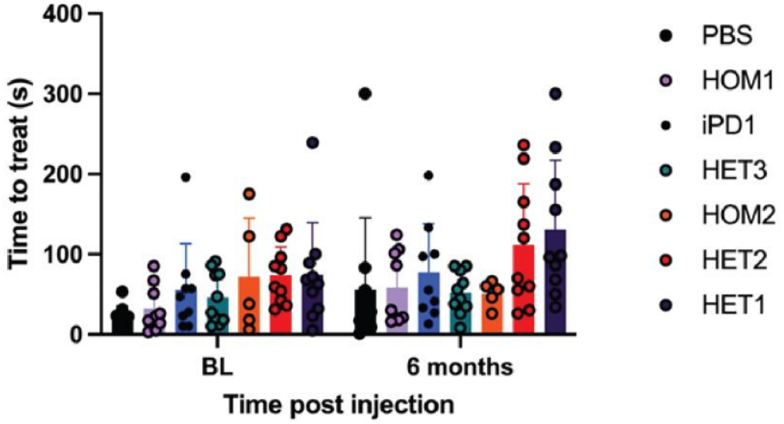

Fig S9: Digging odor test in GBA1$^{D409V/D409V}$ mice treated with different alpha-synuclein human pre-formed fibrils (hPFFs), (A) and wild type mice treated with different alpha-synuclein polymorphs (strains) (B). There was no change in olfactory function observed in the GBA1$^{D409V/D409V}$ mice over the course of 6 months post-injection. This suggests that genotype and WT α-syn fibril injection did not affect olfactory function. There was also no loss of olfactory function over the course of 6 months for the GBA-HuPPF and iPD-HuPFF injected mice, suggesting that α-syn from GBA mutation carriers does not impair olfactory function more than α-syn from iPD patients.

Supplementary Figure 9

A

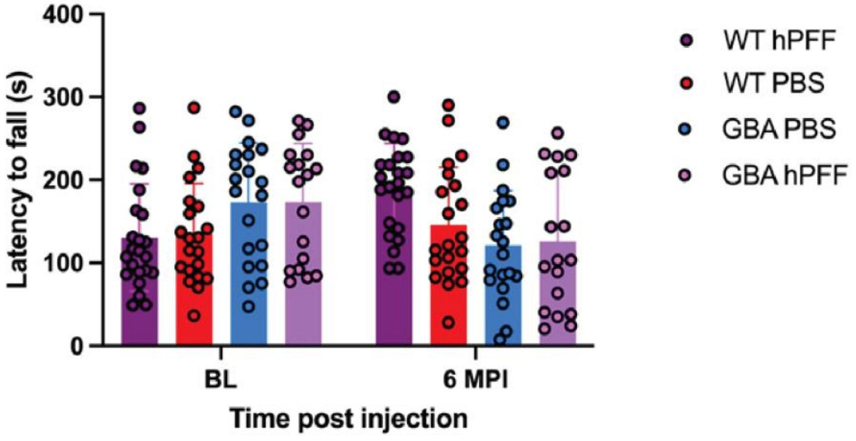

B

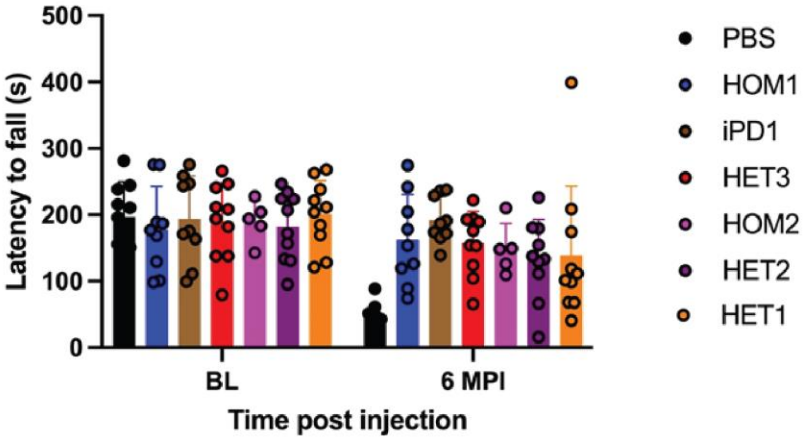

Fig S10: Rotarod test in GBA1$^{D409V/D409V}$ mice treated with different alpha-synuclein human pre-formed fibrils (hPFFs), (A) and wild type mice treated with different alpha-synuclein polymorphs (strains) (B). There was no argument for motor phenotype caused by HuPFFs or genotype based on the accelerating rotarod test. However, as there was no change in the GBA1$^{D409V/D409V}$ mice, it could be inferred that these mice failed to learn how to stay on the rotarod. This could suggest that there was cognitive impairment in these mice.

Supplementary Figure 10

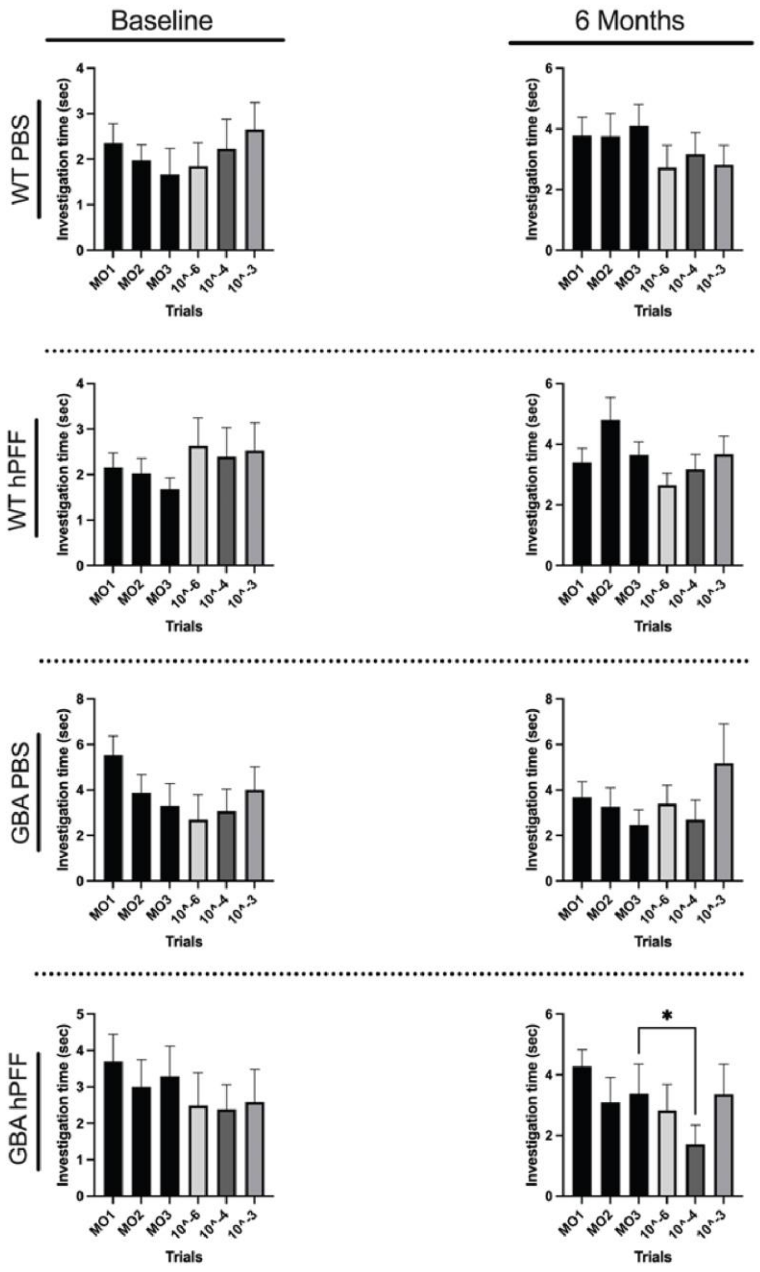

Fig S11: Odor threshold test in GBA1$^{D409V/D409V}$ mice treated with different alpha-synuclein human pre-formed fibrils (hPFFs). At baseline odor threshold, the WT and GBA1$^{D409V/D409V}$ mice that were injected with either PBS or WT-HuPFFs, did not show any ability to detect propionic acid at the three different concentrations (1:10^6, 1:10^4, and 1:10^3). This continued up until 6 months.

Supplementary Figure 11

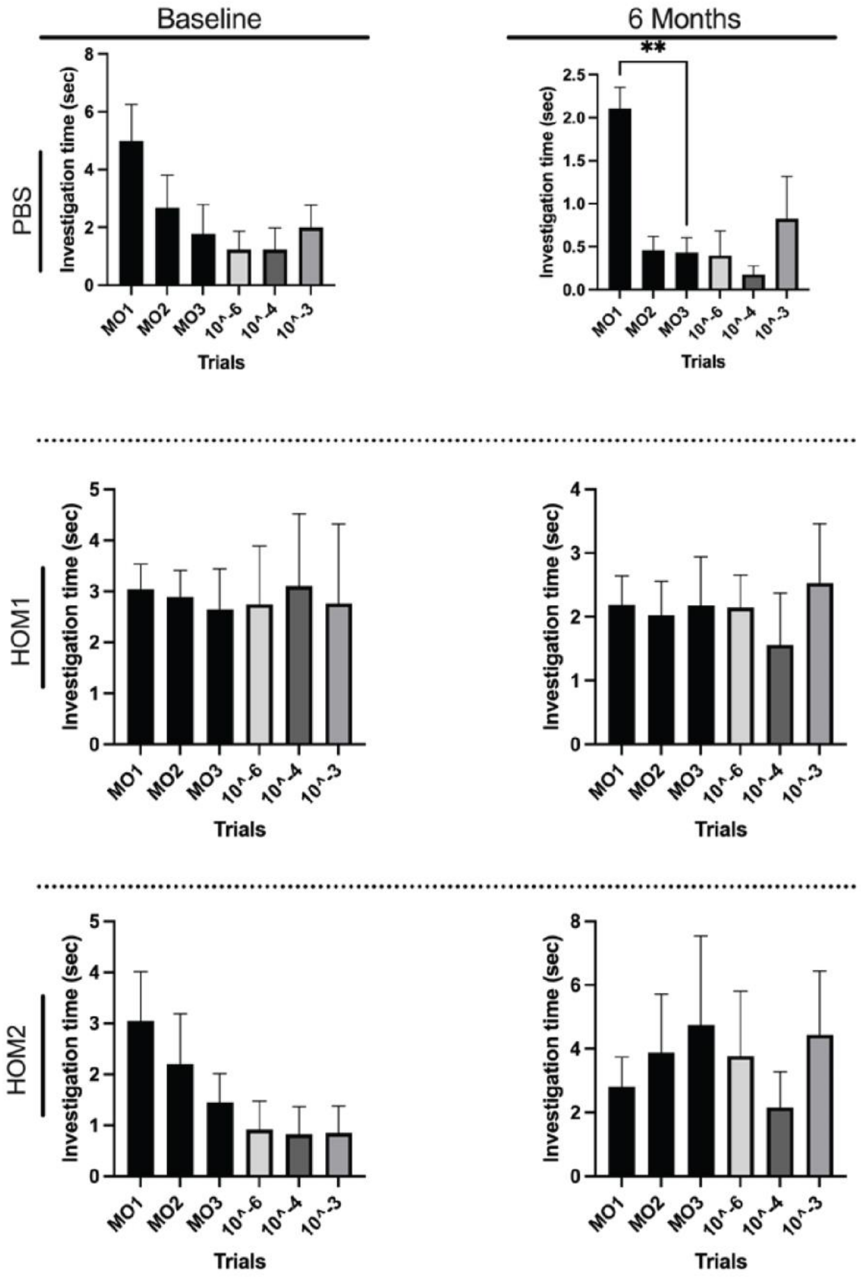

Fig S12: Odor threshold test in wild type mice treated with different alpha-synuclein polymorphs (strains) extracted from the frontal cortex. HOM1 and HOM2 are GBA-HuPPFs. The mice injected with PBS or GBA-HuPPFs did not show any ability to detect propionic acid at the three different concentrations (1:10^6, 1:10^4, and 1:10^3) from baseline to 6 months.

Supplementary Figure 12

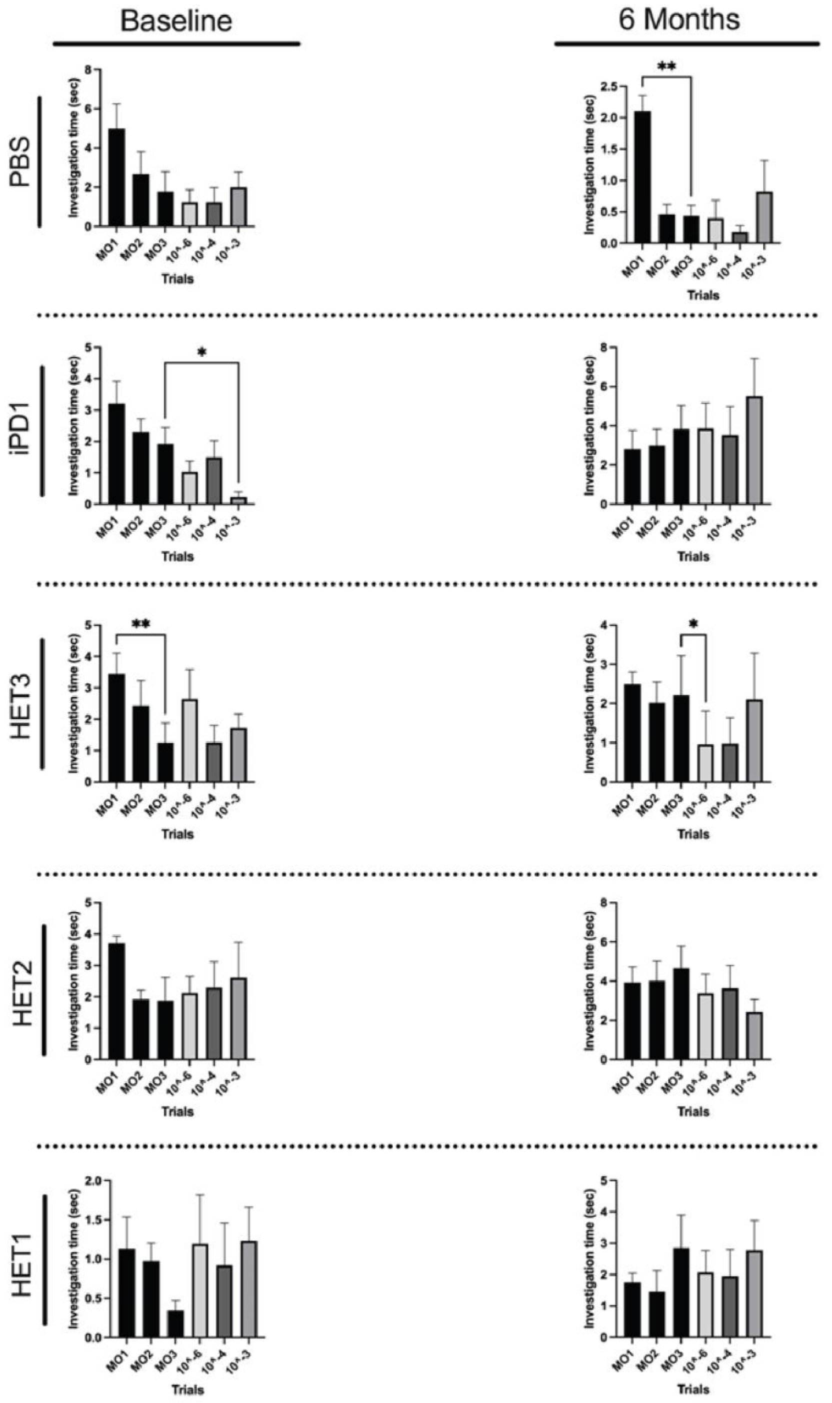

Figure S13: Odor threshold test in wild type mice treated with different alpha-synuclein polymorphs (strains) extracted from the cingulate cortex. The mice injected with PBS, GBA-HuPPFs (HET1, HET2, HET3) or iPD-HuPFFs (iPD1) also did not show any ability to detect propionic acid at the three different concentrations (1:10^6, 1:10^4, and 1:10^3) from baseline to 6 months.

Supplementary Figure 13

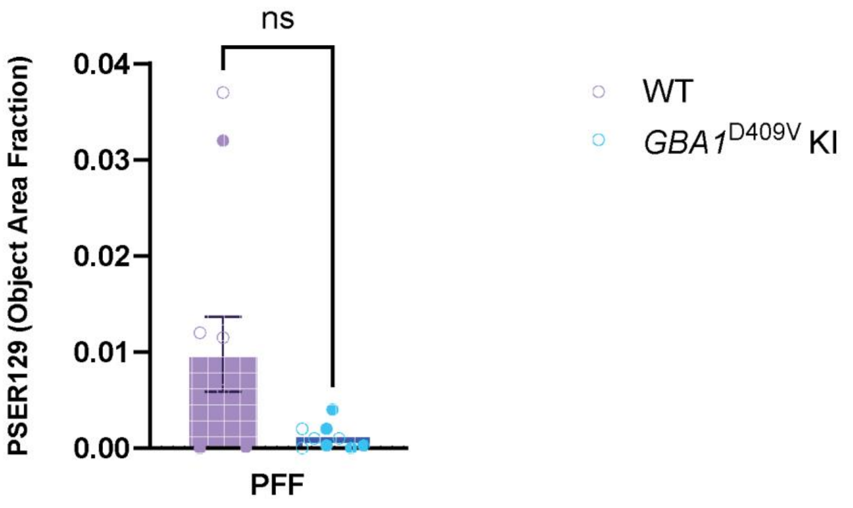

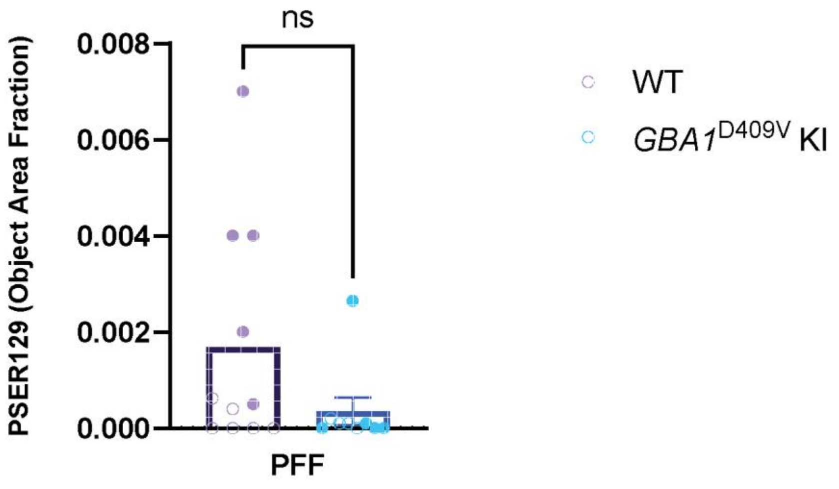

Figure S14. Comparison of PFF spread in WT and *GBA1*^{D409V} KI mice. After removing saline group data from Figure 1, we reanalyzed using two tailed t-test. Statistical significance was not observed both in the olfactory bulb (Two-tailed, t(18)=1.979, p=0.633) or the entorhinal cortex (Two-tailed, t(18) = 1.618 , p=0.1231). ns = nonsignificant

Supplementary Figure 14

Case id	Study Group	Age (y.)	Sex	GBA mutation	Gaucher pathology	Diagnosis	Duration (y.)	Braak Stage	Brain Region
HET1	$GBA^{-/+}$	63	F	H255Q	Unknown	Unknown	Unknown	2	Cingulate
HET2	$GBA^{-/+}$	74	M	E326K	Unknown	Unknown	Unknown	3	Cingulate
HET3	$GBA^{-/+}$	85	F	L444P	Unknown	PD/Dementia	Unknown	Unknown	Cingulate
iPD1	$GBA^{+/+}$	93	M	NA	NA	iPD	12	Unknown	Cingulate
HOM1	$GBA^{-/-}$	61	Female	N370S/c.84insG	Severe	DLB	5	Unknown	Frontal cortex
HOM2	$GBA^{-/-}$	73	Female	N370S/N370S	Minimal	DLB	Unknown	Unknown	Frontal cortex

Supplemental table 1. Case information.

References

1. Mandairon, N., S. Sultan, N. Rey, F. Kermen, M. Moreno, G. Busto,V. Farget, B. Messaoudi, M. Thevenet, and A. Didier. 2009. A computer-assisted odorized hole-board for testing olfactory perception in mice. *J. Neurosci. Methods.* 180:296–303. http://dx.doi.org/10.1016/j.jneumeth.2009.04 .008
2. Rey, N.L., D. Jardanhazi-Kurutz, D. Terwel, M.P. Kummer, F. Jourdan, A. Didier, and M.T. Heneka. 2012a. Locus coeruleus degeneration exacerbates olfactory deficits in APP/PS1 transgenic mice. *Neurobiol. Aging.* 33:426.e1–426.e11. http://dx.doi.org/10.1016/j.neurobiolaging .2010.10.009
3. Rey, N. L. *et al.* Spread of aggregates after olfactory bulb injection of alpha-synuclein fibrils is associated with early neuronal loss and is reduced long term. *Acta Neuropathol* **135**, 65-83, doi:10.1007/s00401-017-1792-9 (2018).
