# Supplementary material for: Neither alpha-synuclein fibril strain nor host murine genotype influences seeding efficacy
Source: NPJ Parkinsons Dis. 2024 May 21;10:105. doi: 10.1038/s41531-024-00679-1 (PMC11109094; doi:10.1038/s41531-024-00679-1)
Supplement: Supplementary file 2 — Related Manuscript File [file 41531_2024_679_MOESM2_ESM.pdf]

nature portfolio

Corresponding author(s): BRYAN KILLINGER

Last updated by author(s): 2-7-2024

Reporting Summary

Nature Portfolio wishes to improve the reproducibility of the work that we publish. This form provides structure for consistency and transparency in reporting. For further information on Nature Portfolio policies, see our Editorial Policies and the Editorial Policy Checklist.

Statistics

For all statistical analyses, confirm that the following items are present in the figure legend, table legend, main text, or Methods section.

n/a	Confirmed	
☐	☒	The exact sample size (*n*) for each experimental group/condition, given as a discrete number and unit of measurement
☐	☒	A statement on whether measurements were taken from distinct samples or whether the same sample was measured repeatedly
☐	☒	The statistical test(s) used AND whether they are one- or two-sided<br>*Only common tests should be described solely by name; describe more complex techniques in the Methods section.*
☐	☒	A description of all covariates tested
☐	☒	A description of any assumptions or corrections, such as tests of normality and adjustment for multiple comparisons
☒	☐	A full description of the statistical parameters including central tendency (e.g. means) or other basic estimates (e.g. regression coefficient) AND variation (e.g. standard deviation) or associated estimates of uncertainty (e.g. confidence intervals)
☐	☒	For null hypothesis testing, the test statistic (e.g. *F*, *t*, *r*) with confidence intervals, effect sizes, degrees of freedom and *P* value noted<br>*Give P values as exact values whenever suitable.*
☒	☐	For Bayesian analysis, information on the choice of priors and Markov chain Monte Carlo settings
☒	☐	For hierarchical and complex designs, identification of the appropriate level for tests and full reporting of outcomes
☒	☐	Estimates of effect sizes (e.g. Cohen's *d*, Pearson's *r*), indicating how they were calculated

Our web collection on statistics for biologists contains articles on many of the points above.

Software and code

Policy information about availability of computer code

Data collection	NIKON ELEMENTS SOFTWARE WAS USED TO QUANTIFY PATHOLOGY.
Data analysis	GRAPHPAD PRISM, R HEATMAP PACKAGE, CYTOSCAPE, GPROFILER, STRING, AND NIKON ELEMENTS SOFTWARE.

For manuscripts utilizing custom algorithms or software that are central to the research but not yet described in published literature, software must be made available to editors and reviewers. We strongly encourage code deposition in a community repository (e.g. GitHub). See the Nature Portfolio guidelines for submitting code & software for further information.

Data

Policy information about availability of data

All manuscripts must include a data availability statement. This statement should provide the following information, where applicable:

- Accession codes, unique identifiers, or web links for publicly available datasets
- A description of any restrictions on data availability
- For clinical datasets or third party data, please ensure that the statement adheres to our policy

All data will be available upon request. Mass spectrometry raw data files are available via PRIDE data repository under the identifier PXD 045437. Detailed protocols for detection of seeded pathology and BAR are made publicly available through dx.doi.org/10.17504/protocols.io.5jyl8pzzdg2w/v1 and dx.doi.org/10.17504/protocols.io.14egn3qq6l5d/v1, respectively.

Research involving human participants, their data, or biological material

Policy information about studies with human participants or human data. See also policy information about sex, gender (identity/presentation), and sexual orientation and race, ethnicity and racism.

Reporting on sex and gender	NA
Reporting on race, ethnicity, or other socially relevant groupings	NA
Population characteristics	NA
Recruitment	NA
Ethics oversight	NA

Note that full information on the approval of the study protocol must also be provided in the manuscript.

Field-specific reporting

Please select the one below that is the best fit for your research. If you are not sure, read the appropriate sections before making your selection.

☒ Life sciences ☐ Behavioural & social sciences ☐ Ecological, evolutionary & environmental sciences

For a reference copy of the document with all sections, see nature.com/documents/nr-reporting-summary-flat.pdf

Life sciences study design

All studies must disclose on these points even when the disclosure is negative.

Sample size	Sample sizes were chosen based on empirical works with the OB-PFF model.
Data exclusions	Mice died prior to study endpoint were excluded from analysis.
Replication	Study was not repeated.
Randomization	NA
Blinding	All Images were gathered and quantified by a reviewer blinded to experimental treatment information.

Reporting for specific materials, systems and methods

We require information from authors about some types of materials, experimental systems and methods used in many studies. Here, indicate whether each material, system or method listed is relevant to your study. If you are not sure if a list item applies to your research, read the appropriate section before selecting a response.

Materials & experimental systems

n/a	Involved in the study	
☐	☒	Antibodies
☒	☐	Eukaryotic cell lines
☒	☐	Palaeontology and archaeology
☐	☒	Animals and other organisms
☐	☒	Clinical data
☒	☐	Dual use research of concern
☒	☐	Plants

Methods

n/a	Involved in the study	
☒	☐	ChIP-seq
☒	☐	Flow cytometry
☒	☐	MRI-based neuroimaging

Antibodies

Antibodies used	EP1536Y (Abcam), Clone 42/α-Synuclein BD Transduction Laboratories.
Validation	We have conducted extensive validation of antibody EP1536Y (Abcam). We have used, preabsorbtion, alpha-synuclien knockout mice, primary delete, and western blot studies to validate our use of this antibody.

Animals and other research organisms

Policy information about studies involving animals; ARRIVE guidelines recommended for reporting animal research, and Sex and Gender in Research

Laboratory animals	57BL/6N-Gba1tm1.1Mjff/J mice (GBA1 D409V KI) and C57BL/6 WT mice from jackson labs.
Wild animals	NA
Reporting on sex	Both sex were used. But no sex specific effects were hypothesized.
Field-collected samples	NA
Ethics oversight	Animal maintenance and experiments were performed in accordance with the National Institutes of Health guidelines and approved by the Institutional Animal Care and Use Committee of the Rush University Medical Center (Chicago, IL)

Note that full information on the approval of the study protocol must also be provided in the manuscript.

Clinical data

Policy information about clinical studies

All manuscripts should comply with the ICMJE guidelines for publication of clinical research and a completed CONSORT checklist must be included with all submissions.

Clinical trial registration	na
Study protocol	na
Data collection	na
Outcomes	na

Plants

Seed stocks	NA
Novel plant genotypes	NA
Authentication	NA
